# Lack of Small Intestinal Dysbiosis Following Long-Term Selective Inhibition of Cyclooxygenase-2 by Rofecoxib in the Rat

**DOI:** 10.3390/cells8030251

**Published:** 2019-03-15

**Authors:** Bernadette Lázár, Gábor B. Brenner, András Makkos, Mihály Balogh, Szilvia B. László, Mahmoud Al-Khrasani, Barbara Hutka, Emese Bató, Eszter Ostorházi, János Juhász, Ágnes Kemény, Terézia László, László Tiszlavicz, Zoltán Bihari, Zoltán Giricz, Dóra Szabó, Zsuzsanna Helyes, Péter Ferdinandy, Klára Gyires, Zoltán S. Zádori

**Affiliations:** 1 Department of Pharmacology and Pharmacotherapy, Semmelweis University, 1089 Budapest, Hungary; lazar.bernadette@med.semmelweis-univ.hu (B.L.); brenner.gabor@med.semmelweis-univ.hu (G.B.B.); makkos.andras@med.semmelweis-univ.hu (A.M.); balogh.mihaly@med.semmelweis-univ.hu (M.B.); laszlo.szilvia@med.semmelweis-univ.hu (S.B.L.); al-khrasani.mahmoud@med.semmelweis-univ.hu (M.A.-k.); hutka.barbara@gmail.com (B.H.); giricz.zoltan@med.semmelweis-univ.hu (Z.G.); peter.ferdinandy@pharmahungary.com (P.F.); gyires.klara@med.semmelweis-univ.hu (K.G.); 2 Second Department of Internal Medicine and Cardiology Center, University of Szeged, 6725 Szeged, Hungary; horeyezonr@gmail.com; 3 Institute of Medical Microbiology, Semmelweis University, 1089 Budapest, Hungary; droeszter@gmail.com (E.O.); szabo.dora@med.semmelweis-univ.hu (D.S.); 4 Faculty of Information Technology and Bionics, Pázmány Péter Catholic University, 1083 Budapest, Hungary; juhaszjanos4@gmail.com; 5 Department of Medical Biology, University of Pécs, 7624 Pécs, Hungary; kemenyagnes1@gmail.com; 6 Department of Pharmacology and Pharmacotherapy, Medical School & Szentágothai Research Centre, University of Pécs, 7624 Pécs, Hungary; zsuzsanna.helyes@aok.pte.hu; 7 Department of Pathology, University of Pécs, 7624 Pécs, Hungary; laszlo.terezia@med.semmelweis-univ.hu; 8 Department of Pathology, University of Szeged, 6725 Szeged, Hungary; tiszlavicz.laszlo@med.u-szeged.hu; 9 Xenovea Ltd., 6726 Szeged, Hungary; info.xenovea@gmail.com; 10 Pharmahungary Group, 6722 Szeged, Hungary

**Keywords:** microbiota, intestinal dysbiosis, inflammatory bowel diseases, cyclooxygenase-2, rofecoxib, enteropathy

## Abstract

Intestinal dysbiosis is linked to numerous gastrointestinal disorders, including inflammatory bowel diseases. It is a question of debate if coxibs, selective inhibitors of cyclooxygenase (COX)-2, cause dysbiosis. Therefore, in the present study, we aimed to determine the effect of long-term (four weeks) selective inhibition of COX-2 on the small intestinal microbiota in the rat. In order to avoid mucosal damage due to topical effects and inflammation-driven microbial alterations, rofecoxib, a nonacidic compound, was used. The direct inhibitory effect of rofecoxib on the growth of bacteria was ruled out in vitro. The mucosa-sparing effect of rofecoxib was confirmed by macroscopic and histological analysis, as well as by measuring the intestinal levels of cytokines and tight junction proteins. Deep sequencing of bacterial 16S rRNA revealed that chronic rofecoxib treatment had no significant influence on the composition and diversity of jejunal microbiota. In conclusion, this is the first demonstration that long-term selective inhibition of COX-2 by rofecoxib does not cause small intestinal dysbiosis in rats. Moreover, inhibition of COX-2 activity is not likely to be responsible per se for microbial alterations caused by some coxibs, but other drug-specific properties may contribute to it.

## 1. Introduction

Over the past decade, it has become increasingly recognized that nonsteroidal anti-inflammatory drugs (NSAIDs), which are among the most commonly used medications worldwide [1], both damage the stomach and duodenum and also injure the lower parts of the gastrointestinal (GI) tract. Small intestinal injury (enteropathy) may occur in up to 30–70% of long-term NSAID users and can manifest in a wide variety of ways, including inflammation, malabsorption, and mucosal ulcers [2,3]. At present, there are no proven ways of either preventing or treating enteropathy. Preclinical studies suggest that antisecretory agents are not only ineffective, but can even exacerbate the intestinal inflammation [4,5].

Since the recognition of NSAID-induced enteropathy, much effort has been put into understanding its pathogenesis and several contributing factors have been identified [6,7,8,9]. One of these factors is the suppression of cyclooxygenase (COX)-mediated prostaglandin (PG) synthesis. COX exists in two isoforms, COX-1 and COX-2. The first is constitutively expressed in the GI tract, whereas COX-2 has little or no expression in most tissues but is rapidly induced by inflammatory and mitogenic stimuli [10]. Therefore, in order to exploit the anti-inflammatory effect of NSAIDs but at the same time avoid their undesired GI side effects, selective inhibitors of COX-2 (coxibs) were developed. According to the original expectations, these drugs produce less gastroduodenal damage than the nonselective NSAIDs [11,12,13,14,15]. However, in the lower segments of the gut, their safety is less obvious. Several studies concluded that chronic treatment with coxibs is associated with much lower incidence of significant intestinal events than treatment with non-selective NSAIDs [16,17,18]. Similarly, it was reported that selective inhibition of COX-2 is likely to be safe in patients with inflammatory bowel diseases (IBDs), at least in the short term [19]. In contrast, there is some evidence that long-term suppression of COX-2 activity, either pharmacologically or through gene inactivation, may induce damage to the intact intestinal mucosa, which is comparable to that caused by the nonselective drugs [20,21,22]. Moreover, coxibs may not only impair mucosal healing mediated by the COX-2 enzyme and exacerbate intestinal inflammation in IBD [22,23,24,25], but may even precipitate de novo colitis [26].

Thus, although the available data are controversial, administration of coxibs, especially long-term, appears to be more frequently associated with intestinal than gastroduodenal complications. One main difference between the luminal environments of the upper and lower GI tracts is the significantly higher abundance of bacteria, which contributes largely to the pathogenesis of NSAID-enteropathy [6,9]. Intestinal bacteria can aggravate NSAID-induced mucosal injury via multiple mechanisms, including impaired ulcer healing and promotion of the enterohepatic recirculation of NSAIDs by deconjugating them [27]. Moreover, it has long been recognized that nonselective NSAIDs can induce small intestinal dysbiosis, in most cases by causing a shift from Gram-positive to predominantly Gram-negative bacteria [28,29,30,31], which is believed to substantially contribute to the development of enteropathy. There is also accumulating evidence that intestinal dysbiosis may predispose to IBD [32]. Regarding coxibs, recent findings suggest that even selective COX-2 inhibitors can change the gut microbiota. Long-term treatment with celecoxib was shown to induce intestinal (ileal and fecal) dysbiosis in mice [33], whereas firocoxib changed the microbiota in horses [34]. Other treatments, however, did not find any microbial alterations in response to celecoxib-treatment [35]. Hence, although the available data are sparse and somewhat inconsistent, prolonged suppression of COX-2 activity may cause intestinal dysbiosis, which could at least partly explain the apparent difference between the safety profiles of coxibs in the upper and lower GI tracts.

It is also important to clarify whether intestinal dysbiosis is caused by inhibition of COX-2 itself (which, in contrast to the classical view, may be expressed constitutively in the GI tract [36,37]), or by other drug-specific properties. For example, celecoxib was shown to exert direct antibacterial effect against Gram-positive strains [38], which may contribute to or be wholly responsible for the observed dysbiotic effect. In addition, COX-2 inhibitors endowed with low pKa values may damage epithelial cells by topical effects (due to interaction with lipid membranes and/or uncoupling of oxidative phosphorylation) and lead to mild inflammation [39], which can cause intestinal dysbiosis via multiple mechanisms [40].

Therefore, in the present study, we aimed to determine the consequences of selective, long-term inhibition of COX-2 on the composition of small intestinal microbiota in the rat, which to our best knowledge has not been addressed before. In order to avoid inflammation-driven bacterial intestinal dysbiosis, we chose rofecoxib as the selective COX-2 inhibitor test compound. Although this drug was already withdrawn from the market, it is a nonacidic compound (pKa is 8.6), in contrast to the weak acids etoricoxib, parecoxib, and lumiracoxib (with pKa values ranging from 4.6 to 4.9) [41,42,43] and lacks any topical mucosal toxicity [44]. In addition, we aimed at first to rule out any potential direct effects of rofecoxib on the growth of bacteria in vitro, which may cause intestinal dysbiosis.

Here, we report for the first time that long-term inhibition of COX-2 by rofecoxib, a nonacidic GI-sparing drug lacking direct antibacterial properties, does not significantly alter the composition of the small intestinal microbiota in rats. These findings argue against a simple COX-2-mediated direct mechanism in the development of intestinal dysbiosis, and suggest that changes in the microbiota in response to some coxibs may be due at least partly to other drug-specific properties.

## 2. Materials and Methods

### 2.1. Animals

Experiments were carried out on male Wistar rats weighing 180–240 g (Semmelweis University, Budapest, Hungary). Animals were housed in a temperature (22 ± 2 °C)- and humidity-controlled room at a 12 h light/dark cycle. Food and water were available ad libitum.

### 2.2. Ethical Considerations

All efforts were made to minimize animal suffering and to reduce the number of animals used in the experiments. All procedures conformed to the Directive 2010/63/EU on European Convention for the protection of animals used for scientific purposes. The experiments were approved by the National Scientific Ethical Committee on Animal Experimentation and permitted by the government (Food Chain Safety and Animal Health Directorate of the Government Office for Pest County (PEI/001/1493-4/2015)).

### 2.3. In Vivo Studies

#### 2.3.1. Study 1. Evaluating the Potency and Selectivity of Rofecoxib for Cyclooxygenase-2 Using the Carrageenan-Airpouch Model

In order to determine the dose of rofecoxib for the chronic study, doses were first assayed in the carrageenan-air pouch model [45]. Briefly, rats were treated intragastrically once daily with rofecoxib (1, 5, and 10 mg/kg) or 1% hydroxyethylcellulose (vehicle) for five days in a volume of 0.33 mL/100 g. On the fifth day, 2 h after the final gavage, 2 mL of a 1% solution of lambda-carrageenan was injected into an air pouch, which was previously induced by injecting twice (on the first and third days of treatment) 10 mL sterile air subcutaneously into the intrascapular area of the rats under isoflurane anaesthesia. Three hours after the injection of carrageenan, the rats were anaesthetized, the pouch fluid was collected by lavage with 1 mL of cold heparin saline, and its prostaglandin E_2_ (PGE_2_) content, which is derived almost entirely from COX-2 [45], was determined by enzyme-linked immunosorbent assay (ELISA), as described below. The gastric mucosal content of PGE_2_, which mirrors mainly the activity of COX-1 [46], was measured in parallel.

In order to prove that once-daily administration of rofecoxib produces significant prolonged inhibition of COX-2 in the rat, an additional group was treated with 5 mg/kg rofecoxib for four days, and carrageenan was applied 24 h after the final gavage.

#### 2.3.2. Study 2. Evaluating the Effect of Long-Term Rofecoxib Treatment on Gastrointestinal Mucosal Integrity and on the Composition of the Small Intestinal Microbiota

Sixteen rats were randomly allocated into two groups with eight rats in each group and were treated intragastrically with either vehicle (1% hydroxyethylcellulose) or rofecoxib (5 mg/kg) in a volume of 0.33 mL/100 g once daily for four weeks. In order to minimize the cage effect [47] (i.e. false positive difference between the microbiota of vehicle- and rofecoxib-treated animals due to housing them in different cages), rats in both groups were divided and housed in 2-2 cages, with four rats per cage. Body weight was measured daily during the course of the treatment. Because both groups served as sham controls for a parallel study (Brenner et al., under publication), on the 29th day, all rats were anaesthetized with pentobarbital (60 mg/kg intraperitoneally) and underwent thoracotomy, but their left anterior descending coronary artery was not occluded (as in the other groups of the parallel study). Rats were ventilated with rodent ventilator (Ugo-Basile, Gemonio, Italy) with 6.2 mL/kg tidal volume at a rate of 69 ± 3 breath/min according to body weight, their blood pressure was continuously monitored in the carotid artery (AD Instruments, Bella Vista, Australia), and their body temperature was maintained at 37 °C with a heating pad.

One-hundred ninety minutes later, the rats were sacrificed and the stomach and small intestine were excised. The content of distal jejunum was quickly collected, snap-frozen in liquid nitrogen, and stored at −80 °C for analysis of microbial composition and luminal pH. The mucosa of the stomach and small intestine was flushed with cold saline and photographed for subsequent macroscopic analysis. The length of the whole small intestine was measured, as another parameter to assess intestinal inflammation [48]. Full-thickness pieces of the distal jejunum were snap-frozen in liquid nitrogen and stored at −80 °C for analyzing the tissue levels of pro- and anti-inflammatory cytokines. Other portions of tissues were fixed in 10% formalin for evaluation of microscopic GI damage.

### 2.4. Macroscopic Evaluation of Gastrointestinal Damage

High-resolution photographs of the gastric and small intestinal mucosa were thoroughly analyzed and scored in blinded fashion, as follows: 0, no visible morphologic alteration; 1, small (1–2 mm) hyperemic area at 1 site; 2, small (1–2 mm) hyperemic areas at 2 or more sites; 3, extensive (>2 mm) hyperemic area at 1 site; 4, extensive (>2 mm) hyperemic areas at 2 or more sites.

### 2.5. Histological Analysis

Samples taken from the antrum and distal part of the small intestine were fixed in 10% formalin, embedded in paraffin, sectioned (5 µm), and stained with haematoxylin and eosin. Digital micrographs were taken by an Olympus BX51 microscope and Olympus DP50 camera. Histological injury was assessed in blinded fashion by two histopathologists in the case of stomach qualitatively. Whereas, in the case of small intestine, histoligical injury was assessed according to the scoring system described by Mantyh et al. [49] with minor modifications (Table 1). The total histological score (ranging from 0 to 9) was calculated based on the sum of partial scores.

### 2.6. Inflammatory Cytokines

The jejunal levels of distinct inflammatory cytokines were measured by either Luminex xMAP technology, or ELISA. Excised and snap-frozen jejunal tissues were pulverized and homogenized according to the manufacturers’ instructions. The total protein concentration of supernatants was determined by using a bicinchoninic acid assay kit (Thermo Scientific Pierce Protein Research Products, Rockford, IL, USA) with bovine serum albumin as a standard.

Milliplex MAP assay based on the Luminex xMAP technology was performed to determine the protein concentrations of interleukin-1β (IL-1β) and interleukin-10 (IL-10) using customized Milliplex Rat Cytokin/Chemokine Magnetic Bead Panel (Merck Millipore, Burlington, MA, USA). The ELISA kit was used to quantify the protein levels of tumor necrosis factor-α (TNF-α) (Invitrogen, Camarillo, CA, USA). Following previous optimizations all samples were tested in a blind fashion and in duplicate, and the results are given in pg/mg of total protein.

### 2.7. Western Blot Analysis of Occludin and Claudin-1

Distal jejunal tissues were homogenized with a TissueLyser (Qiagen, Venlo, The Netherlands) in lysis buffer containing 200 mM NaCl, 5 mM EDTA, 10 mM Tris, 10% glycerine, and 1 μg/mL leupeptin (pH 7.4), supplemented with a protease inhibitor cocktail (cOmplete ULTRA Tablets, Roche, Basel, Switzerland) and PMSF (Sigma, St. Louis, MO, USA). The homogenized lysates were centrifuged twice at 1,500× *g* and 4 °C for 15 min, then the supernatants were collected and their protein concentration was measured by the bicinchoninic acid assay (Thermo Fisher Scientific, Waltham, MA, USA). Equal amount of protein (40 µg) was mixed with Pierce Lane Marker reducing sample buffer (Thermo Fisher Scientific, Waltham, MA, USA), and loaded and separated in a 4–20% precast Tris-glycine SDS polyacrilamide gel (Bio-Rad, Hercules, CA, USA). Proteins were transferred electrophoretically onto a polyvinylidene difluoride membrane (Bio-Rad, Hercules, CA, USA) at 200 mA overnight. Membranes were blocked with 5% nonfat dry milk (Bio-Rad, Hercules, CA, USA) in Tris-buffered saline containing 0.05% Tween-20 (0.05 % TBS-T; Sigma, St. Louis, MO, USA) at room temperature for 2 h. Membranes were incubated with primary antibodies against occludin (ABT 146, 1:2500, Merck Millipore, Burlington, MA, USA) and claudin-1 (ab15098, 1:1000, Abcam, Cambridge, UK) overnight at 4 °C, followed by 2 h incubation at room temperature with appropriate secondary antibodies. GAPDH was used to control for sample loading and protein transfer and to normalize the content of target protein. Signals were detected with a chemiluminescence kit (Bio-Rad, Hercules, CA, USA) by Chemidoc XRS+ (Bio-Rad, Hercules, CA, USA).

### 2.8. Evaluation of Prostaglandin E_2_ Levels

The levels of PGE_2_ in the gastric mucosa and lavage fluid of air pouches were determined by ELISA (Cayman Chemical, Ann Arbor, MI, USA) [46]. Briefly, gastric mucosa was scraped, homogenized in precooled 100% ethanol containing 10 µM indomethacin, and centrifuged at 10,000× *g* for 10 min at 4 °C. Ethanol was evaporated from the supernatants using a vacuum centrifuge, then the residues were resolved in assay buffer and used for determination of PGE_2_. Lavage fluids were centrifuged at 1,000× *g* for 10 min at 4 °C, and PGE_2_ was measured directly from the supernatant.

### 2.9. Antibacterial Activity Assay

The antibacterial activity of rofecoxib was evaluated on a panel of Gram-positive and Gram-negative bacteria with the broth microdilution method according to the EUCAST guideline (www.eucast.org), as previously described [50]. Celecoxib and various antibiotics were used as positive controls. Bacterial strains were grown on COS agar (Columbia agar + 5% sheep blood, Biomérieux, Budapest, Hungary) at 35.5 °C overnight. Appropriate numbers of colonies were suspended in physiological saline in order to reach the density of 0.5 McFarland for inoculation. Stock solutions containing the different substances were prepared with either 100% (celecoxib) or 50% dimethyl sulfoxide (all other substances, diluted with distilled water). These were two-fold serially diluted from 256–0.5 mg/L in cation-adjusted Mueller-Hinton broth (Biolab, Budapest, Hungary) and 100 μL of each dilution was transferred into microplate holes. Inoculation was carried out with 10 μL of each bacterial suspension. Incubation was performed at 35 °C for 24 h and minimal inhibitory concentrations (MICs) were determined visually.

### 2.10. DNA Extraction, PCR Amplification and Sequencing

Bacterial DNA was extracted from 15 mg small intestinal content per sample using the AquaGenomic Kit (MultiTarget Pharmaceuticals, Salt Lake City, UT, USA) and further purified using KAPA PureBeads (Roche, Basel, Switzerland) according to the manufacturer’s protocols. The concentration of genomic DNA was measured using a Qubit 3.0 Fluorometer with Qubit dsDNA HS Assay Kit (Thermo Fisher Scientific, Waltham, MA, USA). Bacterial DNA was amplified with tagged primers (5′-TCGTCGGCAGCGTCAGATGTGTATAAGAGACAGCCTACGGGNGGCWGCAG and 5′-GTCTCGTGGGCTCGGAGATGTGTATAAGAGACAGGACTACHVGGGTATCTAATCC), covering the V3-V4 region of the bacterial 16S rRNA gene [51]. Polymerase chain reactions (PCR) and DNA purifications were performed according to Illumina’s demonstrated protocol (Part # 15044223 Rev. B). The PCR product libraries were quantified and qualified by using High Sensitivity D1000 ScreenTape on TapeStation 2200 instrument (Agilent Technologies, Waldbronn, Germany). Equimolar concentrations of libraries were pooled and sequenced on an Illumina MiSeq platform (Illumina, San Diego, CA, USA) using MiSeq Reagent Kit v3 (600 cycles PE).

Raw sequencing reads per sample (299.200 ± 86.981) were generated, which were demultiplexed and adapter-trimmed using MiSeq Control Software (Illumina). FastQ Toolkit (Illumina) was applied to trim bases at the 3′- and the 5′-end with a quality score less than 30. Reads having mean quality scores less than 30 and shorter than 250 bp were filtered out. The remaining 212.648 ± 68.407 high-quality sequences per sample were aligned and classified by using the Kraken software and its MiniKraken database 20141208 [52].

### 2.11. Determination of Small Intestinal Luminal pH

The content of the distal jejunum and ileum was collected, suspended in ultra-pure water at a ratio of 1:20, and its pH was measured with a 7310 inoLab pH benchtop meter (Xylem Analytics, Weilheim, Germany).

### 2.12. Materials

Rofecoxib [4-(4′-methylsulfonylphenyl)-3-phenyl-2-(5H)-furanone] was purchased from MedChem Express (Sollentuna, Sweden). All other chemicals, unless otherwise stated, were obtained from Sigma-Aldrich (St. Louis, MO, USA).

### 2.13. Statistics

Data are expressed as mean ± SEM. Statistical analysis of the data was performed with Student t test or Mann-Whitney U test (in case of nonparametric values), or with one-way ANOVA (many groups), followed by Holm-Sidak post hoc test. Two-way repeated measures ANOVA was employed to compare the time course of weight losses.

Discriminate taxa between vehicle- and rofecoxib-treated groups were determined using Wald test in DESeq2 (implemented in QIIME) [53,54]. P-values were adjusted for multiple testing by false discovery rate (FDR) using the Benjamini-Hochberg method. Observed species richness and Shannon diversity index were used to estimate the richness and diversity of microbial community in luminal samples. Principal component analysis (PCA) was used for testing the clustering of samples with the same treatment. The differences between the clusters were measured with Hotelling’s T-square test. The calculation of diversity and the PCA analysis were performed in MATLAB programming environment.

In all cases, a probability of *p* < 0.05 was considered statistically significant.

## 3. Results

### 3.1. Rofecoxib Had no Inhibitory Effect on the Growth of Bacteria In Vitro

In order to rule out any potential direct effect of rofecoxib on the growth of bacteria, rofecoxib was first applied at increasing concentrations to different Gram-positive and Gram-negative strains in the broth microdilution assay. As Table 2 and Table 3 demonstrate, rofecoxib (up to 256 mg/L) had no significant inhibitory effect on the growth of any of the bacteria tested. This markedly differed from the effect of celecoxib, which inhibited the growth of the Gram-positive methicillin-sensitive and -resistant *Staphylococcus aureus* and vancomycin-sensitive and -resistant *Enterococcus faecalis* strains with MICs ranging from 32–64 mg/L. Gram-negative bacteria, such as *Acinetobacter baumannii*, carbapenemase- and colistin-resistant *Klebsiella pneumonia*, *Escherichia coli*, and *Pseudomonas aeruginosa*, were not affected by celecoxib.

### 3.2. Rofecoxib Produced Dose-Dependent, Selective, and Long-Lasting Inhibition of COX-2-Mediated Prostaglandin E_2_ Synthesis In Vivo

**Figure 1:**
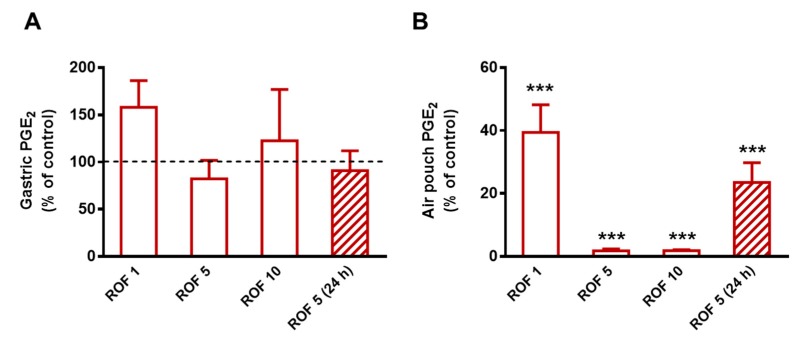
The effect of rofecoxib (ROF, 1 mg/kg, n = 6; 5 mg/kg, n = 8; 10 mg/kg, n = 5) on the levels of PGE2 in the gastric mucosa (**A**) and pouch exudate (**B**) in the carrageenan-airpouch model. The effect of 5 mg/kg rofecoxib was also assayed 24 h after the final gavage (n = 8). The results are expressed as the mean ± SEM percent of the control PGE2 levels measured in vehicle-treated rats. ***p < 0.001 compared to control (one-way ANOVA, Holm-Sidak post hoc test).

Next, we aimed to determine the potency of rofecoxib and confirm its selectivity against COX-2 for the subsequent chronic study. As Figure 1 shows, rofecoxib given for five days and injected 2 h prior to carrageenan on the last day at the lowest dose (1 mg/kg) reduced the concentration of PGE_2_ in the inflammatory exudate by 60.6% (*p* < 0.001), whereas 5 and 10 mg/kg produced an almost complete inhibition of PGE_2_ synthesis (98.2% and 98.1% inhibition, respectively; *p* < 0.001). None of the tested doses affected the gastric mucosal PGE_2_ content significantly. The dose of 5 mg/kg was chosen for the subsequent chronic study, as it proved to be highly effective and selective for COX-2. This dose also corresponds to the maximal recommended daily dose (50 mg) of rofecoxib used earlier in the clinical practice, calculating with a 60 kg weight individual, according to Reagan-Shaw et al. [56].

Although rofecoxib was reported to have a long elimination half-life allowing once-daily dosing [57], we aimed to confirm it by measuring the levels of PGE_2_ 24 h after the final gavage. As the results show, the inhibition of COX-2-derived PGE_2_ synthesis (by 76.5%) remained significant 24 h after the administration of rofecoxib, which allowed once daily administration for the chronic study.

### 3.3. Long-Term Inhibition of Cyclooxygenase-2 by Rofecoxib did not Cause Significant Damage to the Gastrointestinal Mucosa

Although the exact mechanism by which NSAIDs cause intestinal dysbiosis is unknown, mucosal inflammation may alter the microbiota via multiple mechanisms [40], and there is some evidence that chronic inhibition of COX-2 may cause enteropathy [21,22]. Thus, before evaluating the effect of chronic rofecoxib treatment on the composition of microbiota, we aimed to determine whether it had any effect on the GI mucosal integrity.

**Figure 2:**
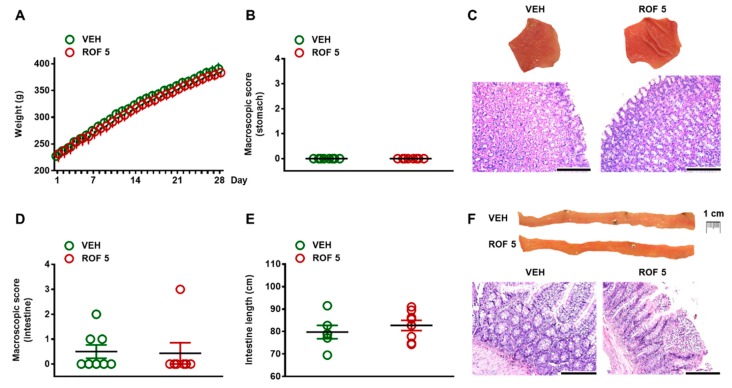
The effects of four-week vehicle (VEH, 1% methylcellulose) and rofecoxib (ROF, 5 mg/kg) treatment on the body weight (**A**) and gastrointestinal mucosa. (**B**): Macroscopic scores of gastric mucosa; (**C**): Representative photos of the gastric mucosa and histological micrographs (haematoxylin-eosin staining); (**D**): Macroscopic scores of small intestinal mucosa; (**E**): Length of small intestines; (**F**): Representative photos of the jejunal mucosa and histological micrographs (haematoxylin-eosin staining), scale bar: 200 µM. There are no signs of any macroscopic or histological tissue damage. (**A**): Results are expressed as the mean ± SEM. Panels (**B**), (**D**), and (**E**): Circles represent the data of each rat, bars indicate the mean ± SEM. For statistical analysis two-way repeated measures ANOVA followed by Holm-Sidak post hoc test (**A**), Mann-Whitney U test (**B**,**D**), and Student’s t test (**E**) were used, n = 8/group.

During the 28 day treatment period, none of the vehicle- or rofecoxib-treated animals died. There was no difference in terms of general condition of animals or weight gain (Figure 2A). As Figure 2B,C demonstrate, the gastric mucosa of rofecoxib-treated rats remained intact and there was no macroscopic sign of any tissue damage. Qualitative histological examination of the mucosa also revealed intact epithelial lining and regular glandular structure.

In the small intestine, thorough macroscopic examination of the entire mucosal surface revealed small hyperemic areas in three of the control rats, whereas an extensive (~1 cm large) hyperemic area was observed in one of the rofecoxib-treated animals. Apart from these, there were no other visible morphologic alterations (like ulcerations, diaphragm-like strictures, ascites, or shortening of the bowel) in any rats (Figure 2D–F). Histological analysis confirmed the macroscopic findings. Although in some rats focal epithelial erosions, mild mononuclear infiltration of the lamina propria or mild edema was observed, they occured in both groups and the overall histological scores of vehicle- and rofecoxib-treated rats were comparable (vehicle: 2 ± 0.3; rofecoxib: 1.2 ± 0.7).

**Figure 3:**
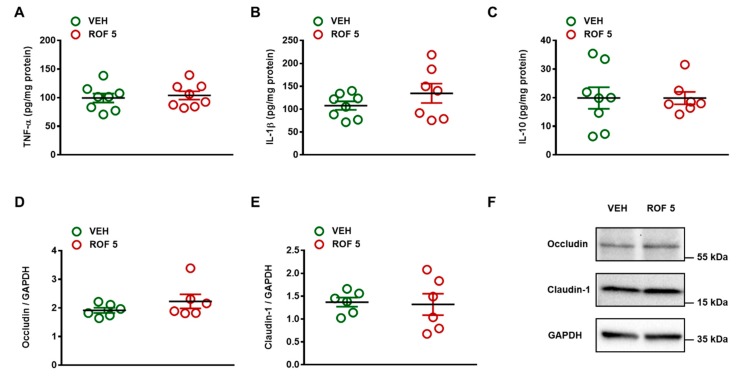
The effect of four-week vehicle (VEH, 1% methylcellulose) and rofecoxib (ROF, 5 mg/kg) treatment on the tissue protein levels of TNF-α (**A**, n = 8/group), IL-1β (**B**, n = 7–8/group), IL-10 (**C**, n = 7–8/group), occludin (**D**, n = 6/group), and claudin-1 (**E**, n = 6/group) in the distal jejunum of rats. Circles represent the data of each rat, bars indicate the mean ± SEM. For statistical analysis, Student’s t test was used. Panel **F**: Representative Western blots for occludin and claudin-1 proteins in the distal jejunum of vehicle- and rofecoxib-treated rats.

The lack of inflammation and tissue damage was also reflected by the unchanged levels of the inflammatory cytokines TNF-α and IL-1β, and the anti-inflammatory cytokine IL-10 (Figure 3). In addition, Western blot analysis revealed similar expression of the tight junction proteins occludin and claudin-1 in vehicle- and rofecoxib-treated animals, suggesting a maintained mucosal barrier function.

NSAID-induced changes in intestinal luminal pH [58] may also lead to dysbiosis because GI pH has a well-recognized role in shaping the microbial community composition [59]. Therefore, we examined whether long-term administration of rofecoxib caused any change in the pH of the jejunal luminal content, but there was no difference between the pH values of the two groups (vehicle: 7.94 ± 0.14, rofecoxib: 7.92 ± 0.13, n = 8/group).

Altogether, these data indicate that the GI mucosa remained intact after four-week treatment with rofecoxib.

### 3.4. Rofecoxib Had no Significant Effect on the Composition of Small Intestinal Microbiota

**Figure 4:**
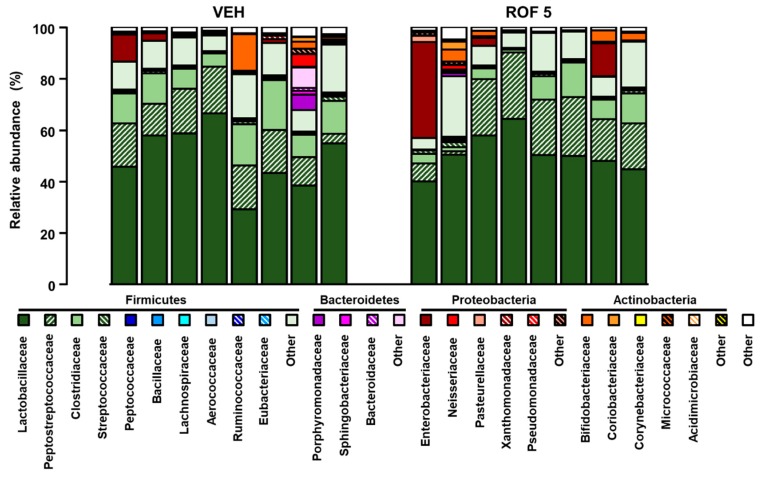
The relative abundance of bacterial families in jejunal samples of rats treated with vehicle (VEH, 1% methylcellulose) and rofecoxib (ROF, 5 mg/kg) for four weeks, determined by deep sequencing of 16S rRNA. Each vertical bar represents the sequencing data for one rat. Unclassified families and families with an abundance less than 0.1% are summarized as “Other”: Relative abundances of the bacterial families were compared between vehicle- and rofecoxib-treated groups by Wald test with Benjamini-Hochberg correction, which did not show any significant difference.

**Figure 5:**
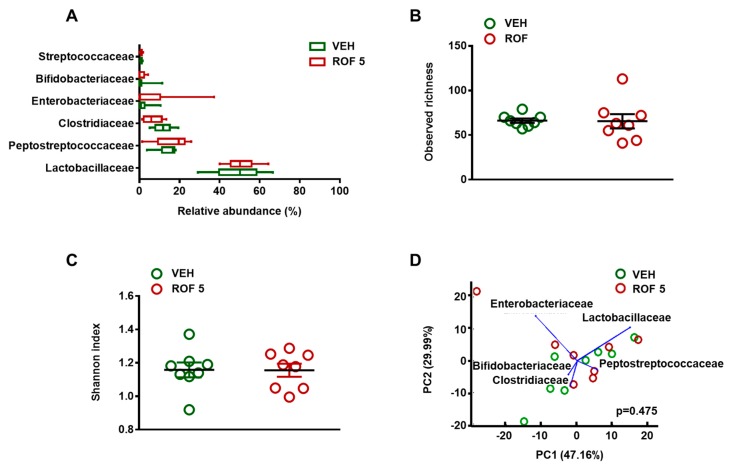
The effects of four-week vehicle (VEH, 1% methylcellulose) and rofecoxib (ROF, 5 mg/kg) treatment on the jejunal microbiota. (**A**): Relative abundances of the most abundant bacterial families in the jejunum. Box and whisker plots indicate the medians, first and third quartiles, and the minimum and maximum values. Panels (**B**) and (**C**): Bacterial richness (observed operational taxonomic units) and diversity estimated by the Shannon index, circles represent the data of each rat, bars indicate the mean ± SEM. (**D**): Principal component analysis (PCA) plot comparing the microbiota composition of vehicle- and rofecoxib-treated rats. The percentage of variation explained by the principal components (PC1 and PC2) is indicated on the axes. There was no clustering between rats treated with vehicle versus rofecoxib. For statistical analysis, Wald test with Benjamini-Hochberg correction (**A**), Mann-Whitney U test (**B**,**C**), and Hotelling’s T-square test (**D**) were used, n = 8/group.

After excluding the possibility of inflammation-driven microbial alterations, the microbiota of vehicle- and rofecoxib-treated animals was determined by deep sequencing of 16S rRNA. At the phylum level, 18 different taxonomic groups were identified. The vast majority of taxa in both the vehicle- and rofecoxib-treated animals belonged to *Firmicutes* (88.8 ± 10 and 87.5 ± 14% of all classified bacteria, respectively), followed by *Proteobacteria*, *Actinobacteria*, and *Bacteroidetes*, whereas the proportion of all other phyla was less than 0.1%. As Figure 4 and Figure 5A demonstrate, the most abundant bacterial family in all samples was *Lactobacillaceae* (control: 49.3 ± 12%, rofecoxib: 50.7 ± 7%), followed generally by two other *Firmicutes* families, *Peptostreptococcaceae* (control: 14.1 ± 5%, rofecoxib: 16.8 ± 8%), and *Clostridiaceae* (control: 11.6 ± 4%, rofecoxib: 6.6 ± 4%). Relative abundances of the identified organisms at all taxonomic levels (from strain to phylum) were compared between vehicle- and rofecoxib-treated groups, but none of the differences reached statistical significance (*p*-values corrected for multiple testing > 0.05). Most abundant bacterial phyla, classes, orders, and genera are shown on Figure S1.

We next examined whether rofecoxib had any effect on the bacterial richness and diversity in the small intestine. There was no difference in the number of observed species and Shannon index between the two groups, indicating similar richness and diversity (Figure 5B,C).

Furthermore, principal component analysis (PCA) failed to identify distinct clusters of microbiota profiles from vehicle- and rofecoxib-treated animals, which further indicates that rofecoxib had no significant effect on bacterial composition (Figure 5D and Figure S2).

## 4. Discussion

This study demonstrates for the first time that long-term (four weeks) selective inhibition of COX-2 by rofecoxib, a compound lacking direct antibacterial and mucosal damaging properties, does not cause small intestinal dysbiosis in rats. These findings suggest that microbial alterations, reported sporadically after repeated administration of some coxibs in different species, cannot simply be explained by inhibition of COX-2 activity and other drug-specific properties may largely contribute to it.

In the present study, we aimed to elucidate whether chronic, selective inhibition of COX-2 has any significant impact on the composition of microbiota. This question was raised since, in some recent publications, intestinal dysbiosis was reported after repeated administration of coxibs. In the study of Montrose et al. [33], a celecoxib-contaning diet (1000 ppm) for 10 weeks decreased the abundance of Lactobacillaceae and Bifidobacteriaceae, whereas the diet increased the abundance of Coriobacteriaceae in the small and large intestine of mice, which was associated with significant alterations of the fecal metabolome and reduced epithelial cell proliferation. Rogers and Aronoff [60] found similar fecal microbial profiles in humans using celecoxib and ibuprofen in the past 30 days, which both were characterized by enrichment of Acidaminococcaceae and Enterobacteriaceae. More recently, temporary changes of fecal microbiota were described in phenylbutazone- and firocoxib-treated horses (drugs were given for 10 days), which were primarily characterized by loss of members of the Firmicutes phylum, specifically the family Lachnospiraceae and, to a lesser extent, the families Clostridiaceae and Ruminococcaceae [34]. In contrast, in a diet-controlled study, celecoxib (200 mg twice daily for 10 days) had no effect on the composition, richness and diversity of fecal microbiome of postmenopausal women [35]. Hence, although the results are contradictory, selective COX-2 inhibitors may evoke bacterial alterations in different species that resemble, in many aspects, those caused by nonselective NSAIDs, such as indomethacin, naproxen, and diclofenac, in both rats [28,31,61,62] and humans [63]. This intestinal dysbiosis is typically associated with a significant decrease in the numbers of Gram-positive bacteria, in favor of Gram-negative microorganisms. Probiotic strains of Gram-positive Lactobacilli and Bifidobacteria can ameliorate intestinal injury by improving the barrier functions and suppressing inflammation [64,65], whereas Gram-negative bacteria can induce a toll-like receptor (TLR)-4-dependent inflammatory reaction [66]. Thus, an altered balance between Gram-positive and Gram-negative bacteria may contribute to the development of both NSAID-enteropathy and other GI diseases, including IBDs [9,32,67]. Thus, revealing microbial alterations caused by selective COX-2 inhibitors is of particular importance, as such effects could at least partly explain the apparent difference between the safety profiles of coxibs in the upper and lower GI tracts. Namely, there is some evidence that these drugs may not only cause relapse of IBD by impairing COX-2-mediated healing processes [23,25], but may also damage the healthy mucosa [21,22,26].

In order to determine whether chronic, selective COX-2 inhibition is associated with any change in the intestinal microbiota, an animal model of enteropathy was used in which rats were treated with rofecoxib for one month. Although rofecoxib was withdrawn from the market owing to serious cardiovascular side effects [68], the rationale behind choosing this compound was that we specifically aimed to exclude any non-COX-2-mediated effects that could potentially influence the outcome of the study.

One such effect is the mucosal damage due to so-called topical effects. It is increasingly recognized that the mucosal damaging effect of NSAIDs is closely related to their acidic and lipophilic chemical structure, which enables these compounds to trigger epithelial cell damage [44]. It was shown that even selective COX-2 inhibitors endowed with acidic character, such as etoricoxib, can cause topical damage to intestinal mucosa [39]. Gut inflammation, on the other hand, can alter the microbiota via multiple mechanisms, including production of reactive oxygen species or nutritional changes, like increased release of ethanolamine from damaged epithelial cells and its subsequent conversion to ammonia [40]. Because rofecoxib is endowed with high pKa (8.6) and does not have topical irritative effect [44], it was expected to be devoid of mucosal damage. Our results confirmed the GI-sparing property of rofecoxib, and showed that it did not cause any significant macroscopic or histological damage to the gastric or small intestinal mucosa. The lack of inflammation was confirmed by unaltered tissue levels of the inflammatory cytokines TNF-α and IL-1β, and the anti-inflammatory cytokine IL-10. In addition, we found that occludin and claudin-1, two tight junction proteins that are localized in the rat jejunum and contribute to barrier properties [69], showed similar expression in vehicle- and rofecoxib-treated animals, suggesting a maintained mucosal barrier function. It is well-established that tight junctions are critical for the maintenance of normal epithelial barrier function and their loss is associated with increased permeability, an important factor in the pathogenesis of both NSAID-enteropathy and IBD [7,70]. Finally, rofecoxib treatment had no effect on the intestinal luminal pH, which has a well-recognized role in shaping the microbial community composition [59], and altered luminal pH caused by some NSAIDs [58] may either be a cause or a consequence of intestinal dysbiosis.

Besides mucosal inflammation also non-COX-2-mediated direct effects on the bacteria may also result in intestinal dysbiosis. Several NSAIDs have been reported to possess direct antibacterial properties, such as ibuprofen, flurbiprofen, ketoprofen and diclofenac [71,72]. Recently, also celecoxib was shown to inhibit the growth of Gram-positive, but not Gram-negative bacteria with MICs ranging from 16–64 mg/L [38], which was also confirmed by our present study. It is hard to estimate whether the antibacterial effect of these NSAIDs makes any meaningful contribution to their dysbiotic effect, as their GI luminal concentrations usually have not been determined. Nevertheless, it can be speculated that NSAIDs undergoing extensive enterohepatic circulation, or those given at high doses, may reach sufficient luminal concentrations to directly affect the growth of distinct bacteria. Such an effect in the case of rofecoxib, however, is unlikely, as it did not influence the growth of various Gram-positive and Gram-negative strains in the broth microdilution assay up to 256 mg/L.

Hence, rofecoxib proved to be a valuable tool for analysing the effect of long-term COX-2 inhibition on the microbiota, as it did not cause GI mucosal damage in vivo and lacked any effect on the growth of bacteria in vitro. As our bacterial 16S rRNA analysis revealed, rofecoxib did not have any major impact on either the composition or diversity of microbiota. The most abundant bacterial phylum in the distal jejunum of rats was Firmicutes, followed by Proteobacteria, Actinobacteria, and Bacteroidetes, which corresponds well to previous reports [62,73]. There were no differences in the proportions of the identified bacterial phyla between control and rofecoxib-treated groups, or in the bacterial proportions at any lower taxonomic levels. The lack of intestinal dysbiosis was also confirmed by comparing the bacterial richness and diversity of the two groups, which were largely similar.

In conclusion, the present study demonstrates for the first time that chronic, selective inhibition of COX-2 by rofecoxib, a compound lacking direct antibacterial and mucosal damaging properties, does not cause small intestinal dysbiosis in rats. Our findings suggest that inhibition of COX-2 enzyme activity is not likely to be responsible per se for microbial alterations caused by some coxibs, and other drug-specific properties, like topical irritancy or direct antibacterial effects, may largely contribute to it. Nevertheless, future studies with different COX-2 inhibitors will allow us to fully understand the effects of coxibs on the intestinal microbiome and also whether dysbiosis caused by COX-2 inhibitors contributes to their potentially harmful effects on the healthy and inflamed gut.

## Figures and Tables

**Table 1:** Criteria for quantitative estimation of the small intestinal injury.

	0	1	2	3
**Epithelial damage**	none	destruction of villus tips	destruction of up to one half of villus	complete villus destruction
**Congestion and edema**	none	minimal increase in crypt spacing, rare RBC-containing vessels	moderate increase in crypt spacing, up to one half of vessels contain RBCs	widely spaced crypts, numerous RBC^1^-containing vessels in lamina propria
**Mononuclear cells**	none	mild mononuclear cell infiltration	moderate mononuclear cell infiltration	numerous mononuclear cells throughout the lamina propria

^1^ RBC–red blood cell.

**Table 2:** Minimum inhibitory concentration (MIC) of celecoxib and rofecoxib against Gram-positive bacteria.

Bacteria	Description	MIC mg/L			
		Rofecoxib	Celecoxib	Vancomycin	Ciprofloxacin
*Staphylococcus aureus* ATCC 29213	Methicillin-sensitive strain (MSSA)	>256	32	1	≤0.5
*Staphylococcus aureus* ATCC 33591	Methicillin-resistant strain (MRSA)	>256	32	1	≤0.5
*Enterococcus faecalis* ATCC 51299	*vanB* vancomycin-resistant strain	>256	64	64	2
*Enterococcus faecalis* ATCC 29212	Vancomycin-sensitive strain	>256	64	2	1

**Table 3:** MIC of celecoxib and rofecoxib against Gram-negative bacteria.

Bacteria	Description	MIC mg/L			
		Rofecoxib	Celecoxib	Imipenem	Colistin
*Acinetobacter baumannii* ATCC BAA1605	MDR strain isolated from the sputum of a Canadian soldier	>256	>256	16	<0.5
*Klebsiella pneumoniae* ST258 clone K 160/09 [55]	Clinical isolate with Carbapenemase (KPC), resistant to carbapenem and colistin	>256	>256	>256	64
*Escherichia coli* ATCC 25218	Quality control strain for susceptibility testing of beta-lactam antibiotics, TEM-1 β-lactamase-producing strain	>256	>256	1	4
*Pseudomonas aeruginosa* ATCC 27853	Quality control strain for E-test Metallo beta-lactamase strip	>256	>256	1	2

## Supplementary Materials

The following are available online at https://www.mdpi.com/2073-4409/8/3/251/s1, **Figure S1:** Relative abundances of the most abundant bacterial phyla, classes, orders and genera in the jejunum of vehicle- and rofecoxib-treated rats. **Figure S2:** Principal component analysis (PCA) plots comparing the microbiota composition of vehicle- and rofecoxib-treated rats at phylum, class, order and genus levels.
