# Supplementary material for: Lack of Small Intestinal Dysbiosis Following Long-Term Selective Inhibition of Cyclooxygenase-2 by Rofecoxib in the Rat
Source: Cells. 2019 Mar 15;8(3):251. doi: 10.3390/cells8030251 (PMC6468807; doi:10.3390/cells8030251)
Supplement: Supplementary file 1 [file cells-08-00251-s001.pdf]

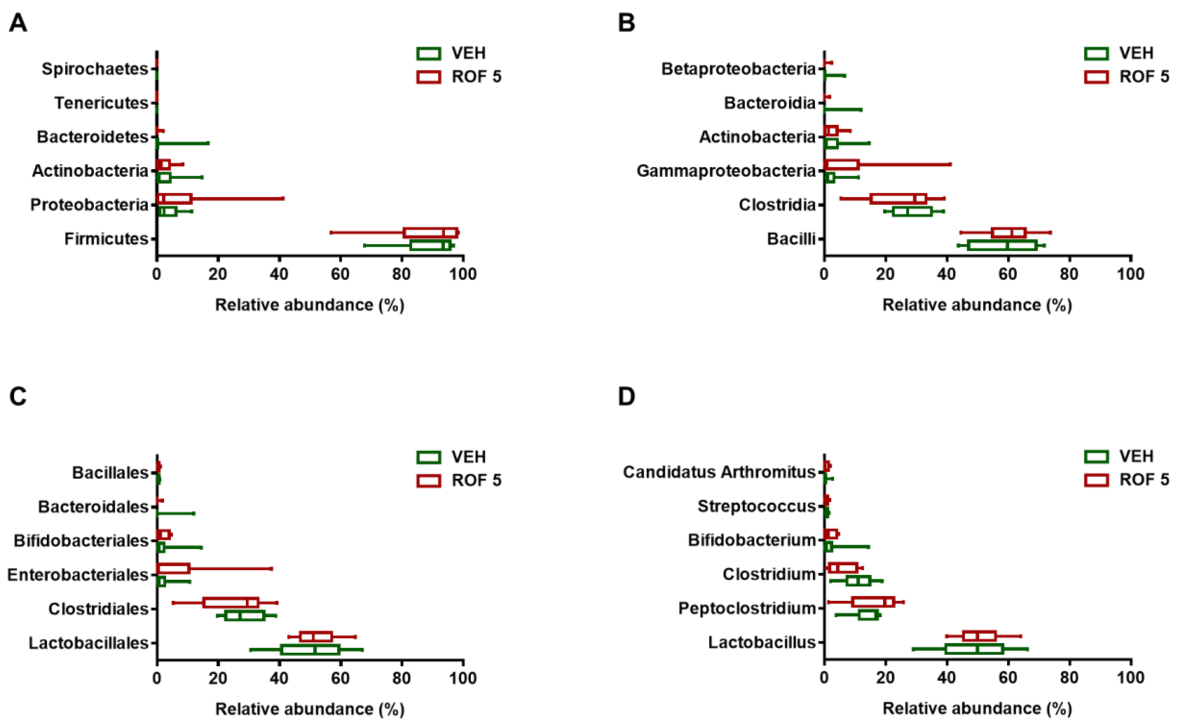

Figure S1. Relative abundances of the most abundant bacterial phyla (A), classes (B), orders (C) and genera (D) in the jejunum of vehicle- (VEH, 1% methylcellulose) and rofecoxib-treated (ROF, 5 mg/kg) rats. Box and whisker plots indicate the medians, first and third quartiles, and the minimum and maximum values. For statistical analysis Wald test with Benjamini-Hochberg correction was used, n=8/group.

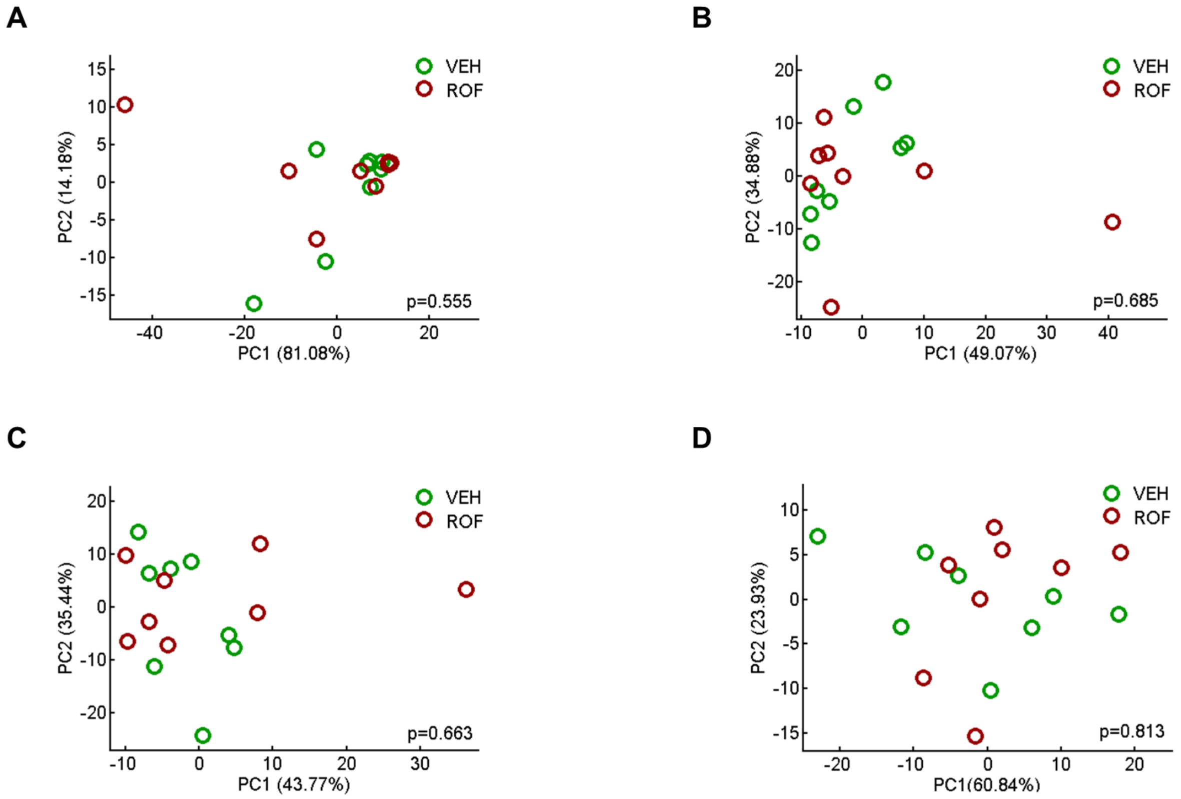

Figure S2. Principal component analysis (PCA) plots comparing the microbiota composition of vehicle- and rofecoxib-treated rats at phylum (A), class (B), order (C) and genus levels (D). The percentage of variation explained by the principal components (PC1 and PC2) is indicated on the axes. There was no clustering

between rats treated with vehicle versus rofecoxib at any level. For statistical analysis Hotelling's T-square test was used, n=8/group.
